# P-373. Effect of Weight Gain on Blood Pressure in Ugandan Persons with HIV on Dolutegravir/Lamivudine/ Tenofovir Disoproxil Fumarate over 48 weeks

**DOI:** 10.1093/ofid/ofaf695.591

**Published:** 2026-01-11

**Authors:** Frank Mulindwa, Willington Amutuhaire

**Affiliations:** United Health Services, Wilson Hospital, Johnson City, NY; Yale School of Medicine, New Haven, Connecticut

## Abstract

**Background:**

Most persons living with HIV in low and middle-income countries are taking fixed dose combination tenofovir disoproxil fumarate/lamivudine/dolutegravir (TLD). Dolutegravir use has been associated with weight gain, a known risk factor for hypertension. There is emerging evidence largely from high income settings, to suggest the observed weight gain with integrase inhibitors correlates to increase in blood pressure. We aimed to determine if similar findings were observed in a cohort of young Ugandan anti-retroviral therapy (ART) naïve patients on TLD over 48 weeks.

**Table 1.**
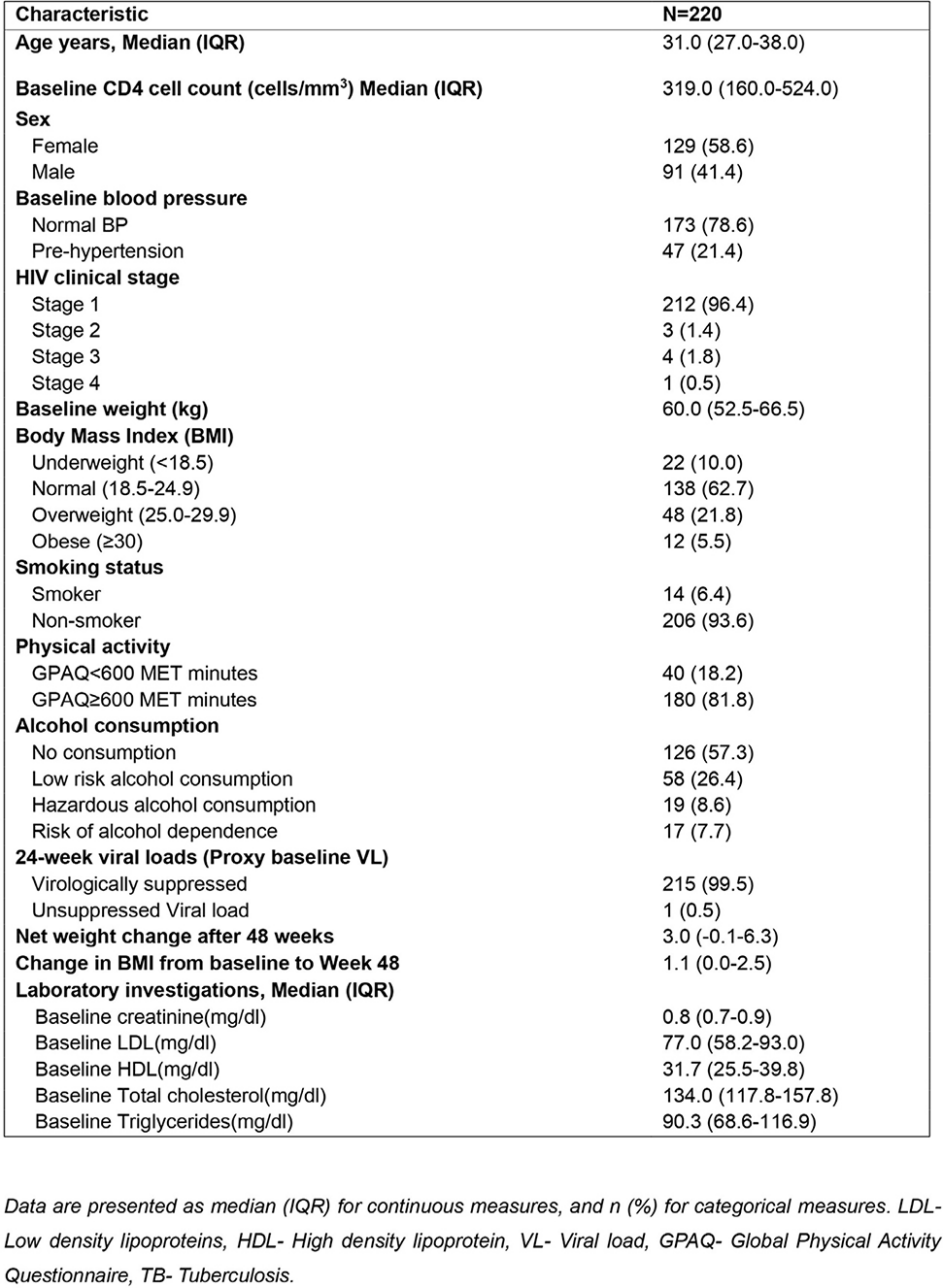
Baseline clinical and demographic characteristics of the study population.

**Figure 2.**
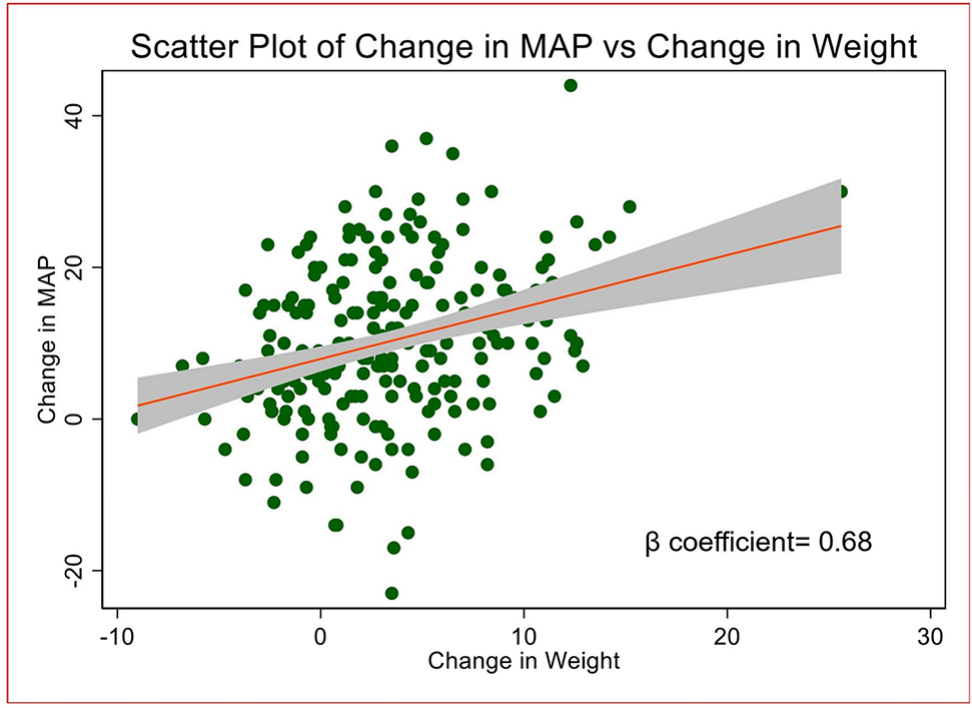
Scatter plot of change in mean arterial pressure versus change in weight with 95% confidence intervals

**Methods:**

We analyzed data from the ‘Glucose metabolism changes in Ugandan PLHIV on Dolutegravir (GLUMED)’ study which was a prospective cohort study with ART naïve persons with HIV ≥ 18 years followed up on TLD over 48 weeks. A scatter plot with 95% confidence intervals and regression line illustrating the relationship between weight change and MAP change from baseline to 48 weeks was created. To further examine the effect of weight change on MAP, we performed a linear regression analysis, with MAP change as the dependent variable and weight change as the independent variable.

**Results:**

Of the 220 patients’ data analyzed, 129 (58.6%) were female, the median baseline age was 31 years (interquartile range (IQR): 27.0-38.0), the median baseline CD4 cell count was 319 cells/mm^3^ (IQR 160.0-524.0). The median weight gain over 48 weeks was 3.0 (IQR: -0.1- 6.3). We found a moderate positive linear relationship between weight gain and MAP over 48 weeks. For every increase in weight of 1kg over 48 weeks, there was an adjusted increase in MAP by 0.62mmHG.

**Conclusion:**

We provide additional evidence to suggest that the noticed weight gain after starting dolutegravir based ART may be associated with a heightened risk of incident hypertension

**Disclosures:**

All Authors: No reported disclosures

